# Modeling interneuron-specific (IS) interneurons in hippocampus

**DOI:** 10.1186/1471-2202-15-S1-P44

**Published:** 2014-07-21

**Authors:** Alexandre Guet-McCreight, Olivier Camiré, Lisa Topolnik, Frances Skinner

**Affiliations:** 1 Toronto Western Research Institute, University Health Network, Toronto, Ontario, M5T 2S8, Canada; 2 Physiology, University of Toronto, Toronto, Ontario, M5S 1A1, Canada; 3 Biochemistry, Microbiology and Bioinformatics, Université Laval, Québec City, Québec, Canada, G1J 2G3; 4 Medicine (Neurology), University of Toronto, Toronto, Ontario, M5S 1A1, Canada

## 

The hippocampus and cortex have a large diversity in inhibitory interneuron types. These local interneurons exert inhibitory control over neuronal populations in the hippocampus [1]. Although much is known about how hippocampal inhibitory interneurons exert control over pyramidal cells and synchronize local network activity, less is known about how the activity of these interneurons are controlled themselves. Moreover, the existence of interneuron-specific (IS) interneurons is known and information about how these IS interneurons exert their influence is accumulating [2]. In particular, interneuron-specific 3 (IS3) cells are being characterized and they have been shown to primarily synapse onto other interneuron dendrites with the ability to control their firing patterns. Morphological and synaptic aspects are being examined, but what type, how much and where voltage-gated channels are present on IS3 has not been determined. As other hippocampal interneuron types are known to have high densities of voltage-gated channels on their dendrites [3,4], it seems likely that this could be the case for IS3 cells also. Determining and understanding particular characteristics of particular cell types is a highly challenging endeavour using purely experimental means. Thus, we have begun development of IS3 computational cell models to help address this challenge.

Using the NEURON software environment [5], we have reconstructed IS3 cell morphologies and built multi-compartment models from them in which appropriate passive properties were obtained. Further, we were able to match several characteristics of representative IS3 cell firings (spike amplitude, spike threshold, rheobase) using four somatically located voltage-gated channel types (sodium current (I_Na_), slow delayed rectifier potassium current (I_Kdrs_), fast delayed rectifier potassium current (I_Kdrf_) and A-type potassium current (I_Ka_). Preliminary data from IS3 cells indicate that they express intrinsic, subthreshold activities similar to other hippocampal interneuron types [6], and previous models captured this aspect with the incorporation of additive white noise to represent stochastic gating characteristics [7]. Similarly here, to capture these aspects, we somatically injected white noise current into our models and found that output similar to experimental data was observed. Given the present correspondence with data, our models already make predictions about channel types and balances found in IS3 cells. Thus our present multi-compartment IS3 models represent a solid basis for determining and understanding the biophysical contributions for IS3 cell firings and their ability to control inhibitory cells and functionally contribute to the generation of hippocampal oscillations.
